# Methods for the development and testing of polymeric hybrid photovoltaic thermal (PVT) collector for indoor experiments

**DOI:** 10.1016/j.mex.2019.10.021

**Published:** 2019-10-23

**Authors:** Erkata Yandri

**Affiliations:** Graduate School of Renewable Energy, Darma Persada University, Jl. Radin Inten 2, Pondok Kelapa, East Jakarta 13450, Indonesia

**Keywords:** Lab-made polymeric PVT collector, Polymeric material, PMMA, PVT collector, Indoor testing, The method for development PVT collector, The method for testing PVT collector

## Abstract

The purpose of this article is to present the methods for the development and testing of polymeric hybrid Photovoltaic Thermal (PVT) collectors for indoor experiments. Polymeric material has been used in hybrid PVT collectors and even longer used on flat plate collectors. We have developed a method to make a hybrid PVT collector with an absorber made of a polymethyl-methacrylate (PMMA) and combined with a copper sheet. To understand the details of the thermal and electrical performance, we have developed a method to test this PVT collector by conducting a series of experiments by varying the mass flow rate, irradiance, and inlet water temperature. The results showed that PMMA as a thermal absorber with a copper sheet can provide a beneficial cooling effect of around 80% thermal efficiency and a 0.03 %/^o^C decrease in electrical efficiency.

• This method makes PVT collector hybrids easily and cheaply using simple equipment.
• This method provides simple technical testing of hybrid PVT collectors
• This method can be applied to make a larger hybrid PVT collector with modular.

## Highlights

• Polymethyl-methacrylate (PMMA) can be used as a thermal absorber for hybrid photovoltaic collector.
• This polymeric hybrid PVT collector can be developed and tested with a simple method.
• The thermal efficiency can achieved be around 81 % and the reduction of electrical efficiency 0.003 %/^o^C.

## Graphical abstract

**Figure:**
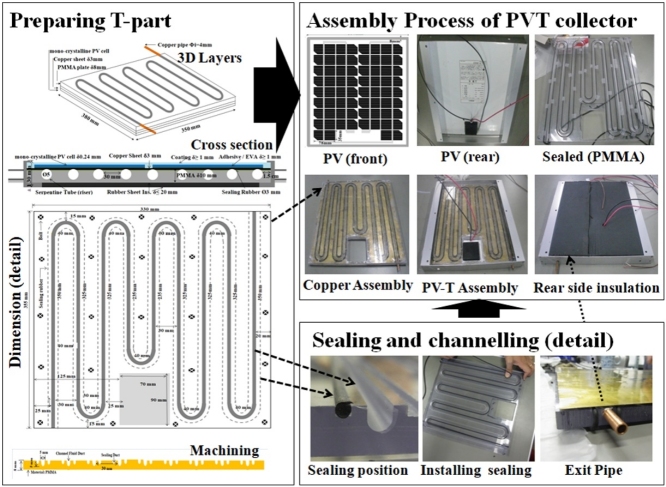

**Specification Table**

**Table:** 

Subject Area:	Energy
More specific subject area:	Polymeric Material for Solar Energy Application
Method name:	Lab-made polymeric PVT collector.
Name and reference of original method:	Development and experiment on the performance of polymeric hybrid Photovoltaic Thermal (PVT) collector with halogen solar simulator: https://www.sciencedirect.com/science/article/pii/S0927024819303952
Resource availability:	The data are available in this article.

## Method details

Solar energy can be converted into heat energy and electrical energy which can be carried out by a hybrid Photovoltaic (PV) and Thermal (T) collector, or PVT collector. PVT collector is very interesting because, with the same aperture area as a flat plate solar collector (FPSC), it can convert more solar energy. PVT collector is a combination of two different solar energy conversion devices, such as PV panels to produce electrical energy and thermal collectors to produce thermal energy. Because the PV panel performance decreases with increasing surface temperature [1], the thermal collector part functions as a heat absorber from solar energy radiation that hits the PV panel surface. This heat retrieval can reduce the surface temperature of the PV panel. As a result, the electrical output becomes more optimal and the heat energy collected can be utilized in other forms.

PVT collector can be applied in residential and industry [2,3]. Since the publication of the first PVT model in 1978 by Florschuetz [4], research on PVT collectors have been carried out intensively. PVT collector has been studied from various aspects with many review papers, such as; historical development and improvement [[5], [6], [7], [8], [9]], general and technological applications [[10], [11], [12], [13], [14]], method of fabrication and integration [15,16], performance analysis with simulations and experiments [17], economical assessment [18], environmental issues [19], and etc. As seen for the PVT collector fabrication and integration method, there are only 2 review papers available online. Wu J. et al. [16] conducted a critical review of thermal absorbers and their integration methods into currently-available PV modules for the purpose of developing combined PVT modules. Abdelrazik AS, et al. [15] conducted a critical review on the construction of PVT collector to the absorber plate with the mechanical and chemical attachment method.

Aste et al. [20] developed the hybrid PVT collector as an “upgrade’’ of a conventional solar air collector, i.e. a front cover direct flow PVT collector, organized in a modular structure. Kumar and Tiwari [21] fabricated a single slope photovoltaic/thermal (PVT) active solar still and tested at solar energy park, IIT New Delhi (India) for the composite climate. Zang et al. [22] designed and fabricated a novel solar photovoltaic/loop heat pipe (PV/LHP) module-based heat pump system for both electricity and hot water generation. Chen and Yin [23] developed and constructed a building integrated photovoltaic-thermal (BIPVT) multifunctional roofing to harvest solar energy in the form of PV electricity as well as heat energy through the collection of warm water. Michael et al. [24] developed the PVT module without any mechanical welding and tested in outdoor conditions that the electrical efficiency of the PV module and PVT module showed similar performance. The details of the fabrication and integration method for the PVT collector are summarized in Table 1 below.

**Table 1:** Summary studies of using non-polymeric material for hybrid PVT collector (more focus on a method for PVT collector).

Items		Aste et al. [20]	Kumar and Tiwari [21]	Zang et al. [22]	Chen and Yin [23]	Michael et al. [24]
PVT type		PVT	PVT/Still	PV/LHP	BIPVT	PVT
PV-part	Type of PV	p-Si	1.2	–	m-Si	p-Si
	Area (m^2^)	1.03	–	0.612	0.26	≈0.51
T-part	Type	–	–	–	Sheet-pipe	Sheet-pipe
	Area (m^2^)	2.4	2 × 2	–	0.37	0.51
	Absorber used	–		Aluminum	Aluminum/HDPE	Copper
	Working Fluid	Air	Water	R1341a	Water	Water
	System	Active	Active	Active/HP	Active	Active
Methods Process	Design/Preparation	–	–	√	√	–
	Integration	–	–	√	√	–
	Testing / simulation	√	√	√	√	√

Type of PV: m-Si (mon-silicon), p-Si(poli-silicon)PVT/LHP: Photovoltaic thermal / loop heat pipeBIPV: Building integrated photovoltaic.

The symbol “- “means the data / information is not available on paper, both written and unwritten.

The symbol “√ “means the data / information is not available on paper, both written and unwritten.

To further promote and increase the competitiveness of PVT collectors, various efforts have been made for economic affordability, such as lowering material cost [25], parameter optimization [26]. In this case, the use of plastic or polymer materials is very important. Material experts assigned by the International Energy Agency in Task-39 on SH-TCP, have completed the task of studying polymer materials for solar energy applications [27]. Herrando et al. [28] proposed to improve PVT collectors with an optimal balance of energy efficiency, weight/strength, cost and ease of manufacture, while maximising heat transfer and the overall efficiency of the collectors.

The polymeric material has been used in hybrid PVT and FPSC, generally for solar energy applications [29]. Many polymers are transparent and can replace the glass. However, polymers normally degrade more easily than glass and hence have fewer applications [30]. Sandness and Rekstadt [31] combined a polymer solar heat collector with single-crystal silicon PV cells in a hybrid energy-generating unit, that simultaneously produced low-temperature heat and electricity and tested experimentally to determine its thermal and photovoltaic performance. Erdil et al. [32] constructed a hybrid PVT system from a photovoltaic (PV) module and a solar thermal collector and tested for energy collection at a geographic location of Cyprus. Christofari et al. [33] studied the thermal behaviour of a solar water heating system using a hybrid PVT collector, based on the polymeric absorber. Ango et al. [34] numerically investigated the thermal behaviour of a polymeric flat plate solar collector (FPSC), to evaluate the influence of various operating and design parameters on the polymer FPSC’s performance. Peña et al. [35] designed and manufactured a polymeric solar water heater with satisfying necessary technical requirements for Mexican homes. Reiter et al. [36] developed a dynamic flat-plate collector model for parametric sensitivity studies on polymeric collector design and showed satisfying results especially regarding the calculation of individual part temperatures of a collector validation. Kroiß et al. [37], developed a sea waterproof hybrid PVT system with the aim of low cost and high electrical/thermal performance. The low-cost was achieved by the adoption of standard components combining a polypropylene thermal absorber with a commercial PV system. It was highlighted that polypropylene shows certain advantages in comparison to established absorber materials (e.g. copper or aluminum). Chen et al. [38,39] studied the environmental influence between traditional and polymeric solar collectors using the methodology of Life Cycle Assessment, found that the environmental influence for the polymeric collector was smaller than that for a traditional solar collector. Afzanizam et al. [40] investigated the integration of low thermal conductivity into a polymeric PVT collector and showed that the polymeric collector is capable of replacing conventional copper and aluminium, with an acceptable range of performances and the additional advantages of ease of handling, manufacturability, lightweight, etc. Asmussen S V, Vallo CI [41] studied the synthesis of polymeric absorber for low-temperature solar collectors (≤100 °C) which consists of a plate to absorb solar radiation and tubes containing a heat-transport fluid to remove heat from the absorber. For a more detailed review, see Table 2 below.

**Table 2:** Summary studies of using polymeric material for hybrid PVT collector (more focus on polymeric material).

Items		Sandness and Rekstadt [31]	Erdil et al. [32]	Christofari et al. [33]	Ango et al. [34]	Peña et al. [35]	Reiter et al. [36]	Kroiß et al., [37]	Chen et al. [38,39]	Afzanizam et al. [40]
Collector type		PVT	PVT	PVT	FPSC	FPSC	FPSC	FPSC	FPSC	PVT
PV-part	Type of PV	m-Si	–	p-Si	–	–	–	p-Si	–	p-Si
	Area (m^2^)	0.32	1.2	0.37	–	–	–	1.46	–	–
T-part	Type	Solarnor	–	Honeycomb	Honeycomb	–	Sheet-pipe	Honeycomb	Honeycomb	Sheet-pipe
	Area (m^2^)	0.48		2.0	0.91	1.86	16 & 24	1.38	4,6,8.10	2.97
	Polymer used	PPO	Tedlar	PC	PC	PP	Polymeric	PP	PC	Polymeric
	Working Fluid	Water	Water	Water	Water	Water	Sheet-pipe	Sea Water	Water	Water
	System	Active	Active	Active	Active	Passive	Passive	Active	Active	Active
Methods Process	Design/Preparation	–	–	–	–	–	–	–	–	–
	Integration	–	–	–	–	–	–	–	–	–
	Testing / simulation	√	√	√	√	√	√	√	√	√

Type of PV: m-Si (mon-silicon), p-Si(poly-silicon).

Polymer used: PPO (Polyphenylen Oxie), PC (Polycarbonate), PP (Polypropylene).

The symbol “- “means the data / information is not available on paper, both written and unwritten.

The symbol “√ “means the data / information is not available on paper, both written and unwritten.

As summarized in Table 1, Table 2, it seems that there is no literature specifies the methodological process of making a hybrid polymeric PVT collector ranging from design/preparation, fabrication and testing. In addition, we have to agree that there are some challenges that must be faced by hybrid polymeric PVT collectors in the future, such as; easy in the manufacturing process, cheap in production costs, and the least impact on the environment, but provides high performance. For that reason, we have developed a polymeric hybrid PVT collector, made of polymethyl-methacrylate (PMMA) and combined with a copper sheet as an absorber [42]. The copper sheet with high thermal conductivity was utilised to simplify the manufacturing process. In addition, the copper sheet can also slow down the degradation process of polymeric material due to direct light exposure continuously [43]. This PMMA PVT collector is as a complement to our indoor PVT experiments with a compact halogen solar simulator [44]. As recommended by the IEA [27], PMMA as a polymeric material, has excellent characteristics as an important component of solar energy technology.

The current work is part of our project to analyse the Joule heating effect of the PVT collector during electricity generation [45]. The experiments have been done in the Solar Energy Laboratory, Kanagawa Institute of Technology, Atsugi, Japan. Therefore, the aim of our paper is to detail the method of design, fabrication/integration and testing performance of polymeric hybrid PVT collectors for indoor experiments. The method of fabricating a polymeric PVT collector needs to be explained systematically, making it easy to duplicate and further develop.

## Methodology

### Development method

#### Preparation PV-part

**Fig. 1:**
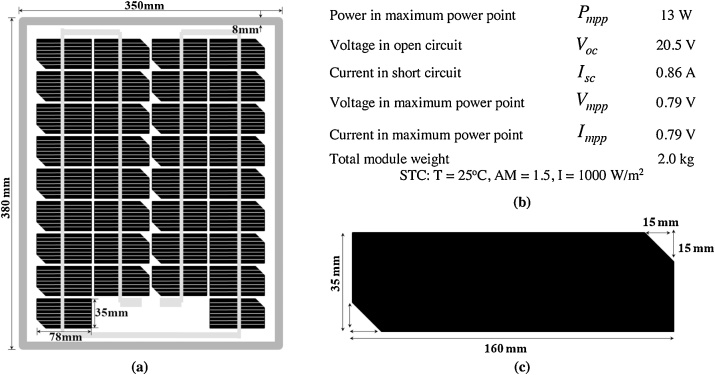
PV module, a) PV module dimension, b). PV module specification, c). The dimension of a segment of the PV module.

The purpose of this process is to prepare the PV-part as the basic construction frame of the PVT-collector. Fig. 1 shows the PV-part of the polymeric PVT collector. As shown in Fig. 1(a), we used the mono-crystalline type PV module (GT434 type, KIS Solar Japan), with outer dimensions of 380 mm × 350 mm and frame thickness of 35 mm. The specification of the PV module can be seen in Fig. 1(b). There are 17 PV cell segments and its dimension as shown in Fig. 1(c). The effective area of PV-part $A_{pv}$ is 0.115 m^2^, which is calculated from:

(1) $$A_{pv}=\sum_{1}^{n}A_{pv,1}+.....+....A_{pv,n}$$

where, n is the number of segments in a PV module. As a result,$A_{pv}$ is 0.091 m^2^. According to Chow et al. [46], the PV packing factor expressed as:

(2) $$\xi =A_{pv}/A_{pvt}$$

Based on the previous data, ξ = 0.791

#### Preparing T-part

**Fig. 2:**
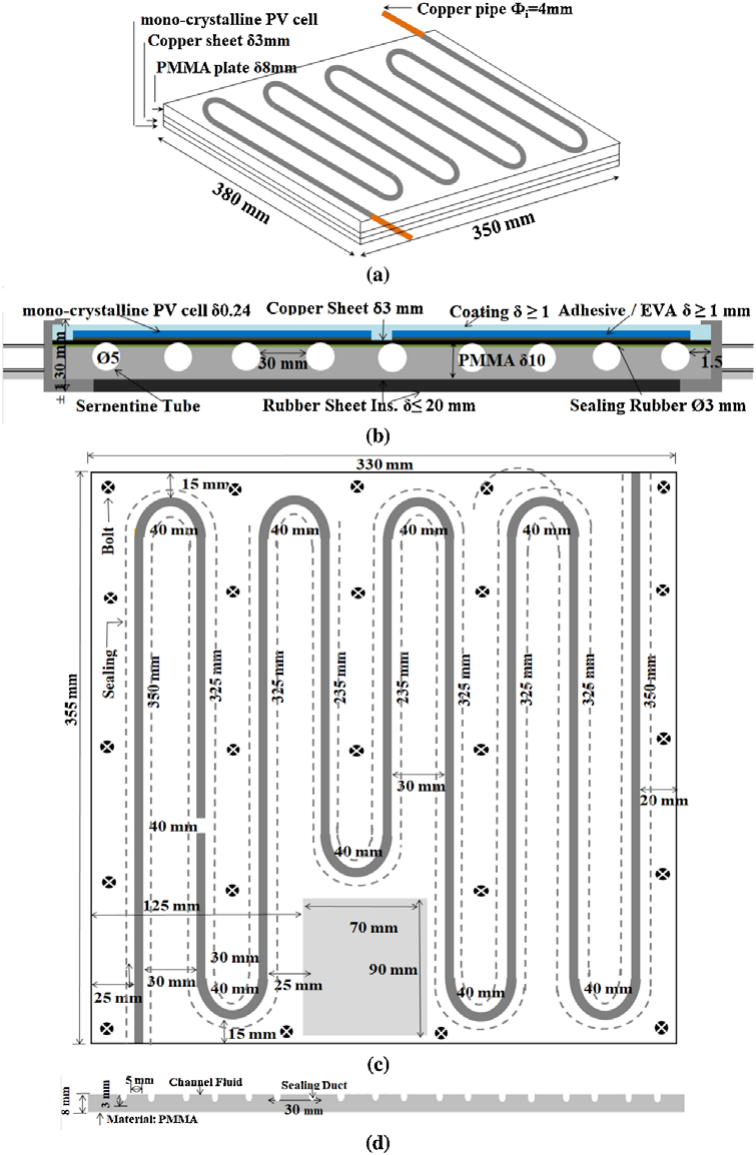
Preparing T-part: a). 3D layers, b). Cross-section view, c). The dimension of T-part, d). Machining of T-part.

The purpose of this process is to prepare the T-part that will be inserted into the PV-part to become a PVT collector. Fig. 2 shows the preparation process of T-part. Fig. 2(a) shows the 3-dimensional layer-by-layer polymeric hybrid PVT collector and Fig. 2(b) shows the layered structure of the PVT collector in a cross-sectional view. The frame of PV-part was used as the basic construction, to become a PVT collector with the complete layers; PV cell, copper sheet, adhesive/ethylene vinyl acetate (EVA), PMMA absorber, and rubber sheet insulation. The frame of the PV module facing the wiring box must be opened in preparation for the inserting direction of the T-part. The frame is opened by carefully scraping the sealing and opening the screw. Since the beginning of the design, it must be carefully calculated between the channel flow to provide the optimal and uniform cooling effect.

Due to our limitations in T-part fabrication, we have covered the open channel with a δ3 mm thick copper sheet. Consequently, the use of this copper sheet can create additional resistance that reduces thermal efficiency [31]. Then, from the surface of the copper sheet, some metal bolts are inserted a few mm into the PMMA plate to prevent leakage. The copper sheet of the absorber as the reference collector has a low emission coefficient (ε = 0.019) in comparison to the emission coefficient of the polymeric absorber (ε = 0.95). Previously, we had tried a copper plate with a thickness of 0.3 mm which was bonded with EVA, but often leaked. For connection with the external hose, the channel inlet-outlet sections are fitted with a copper pipe Ø_i_4 mm. To simplify the manufacturing process, the serpentine type channel adjusted the shape and size of the PV module for more heat absorption and increase the thermal performance [[47], [48], [49]].

Fig. 2(c) shows the serpentine type channel dimensions of the T-part, the polymeric plate of 355 mm x 330 mm has a thickness of 10 mm. To adjust the construction, dimensions, and function of the PV-part, the total length of the one-way channel is 2795 mm. The distance between the two outer walls of the channel is 30 mm. Thus, it is expected that each PV cell will get the optimum cooling effect. Along with the channel sealing, rubber strings of Ø3 mm were installed to be impermeable and leak-proof. In order for the sealing rubber to function optimally, the installation must be done carefully. Do not get too pulled because it will reduce the flatness of the surface that can cause leakage. A silicon adhesive ethylene vinyl acetate (EVA) with a thickness of δ3 mm was used sufficiently to absorb the difference in thermal expansion between the PMMA absorber and the copper sheet, ensuring the acceptable thermal contact and improving the transmittance of the absorber [50]. Between the copper sheet of the T-part and the plastic sheet of PV-part, there is direct contact without using EVA. To withstand heat losses, the back part of collector PVT was isolated with a *δ*10 mm rubber sheet. Fig. 2(d) shows an open channel of 5 mm in width and 5 mm in depth was machined. The width of the serpentine channel is made uniform from the inlet to the outlet to reduce the resistance experienced by water as a working fluid. It is important to note that there is no sanding process on the PMMA sheet surface. PMMA is considered flat to be attached to a copper sheet and then tightened with bolts: refer to Fig. 2(c).

#### Integration PV-part and T-part

**Fig. 3:**
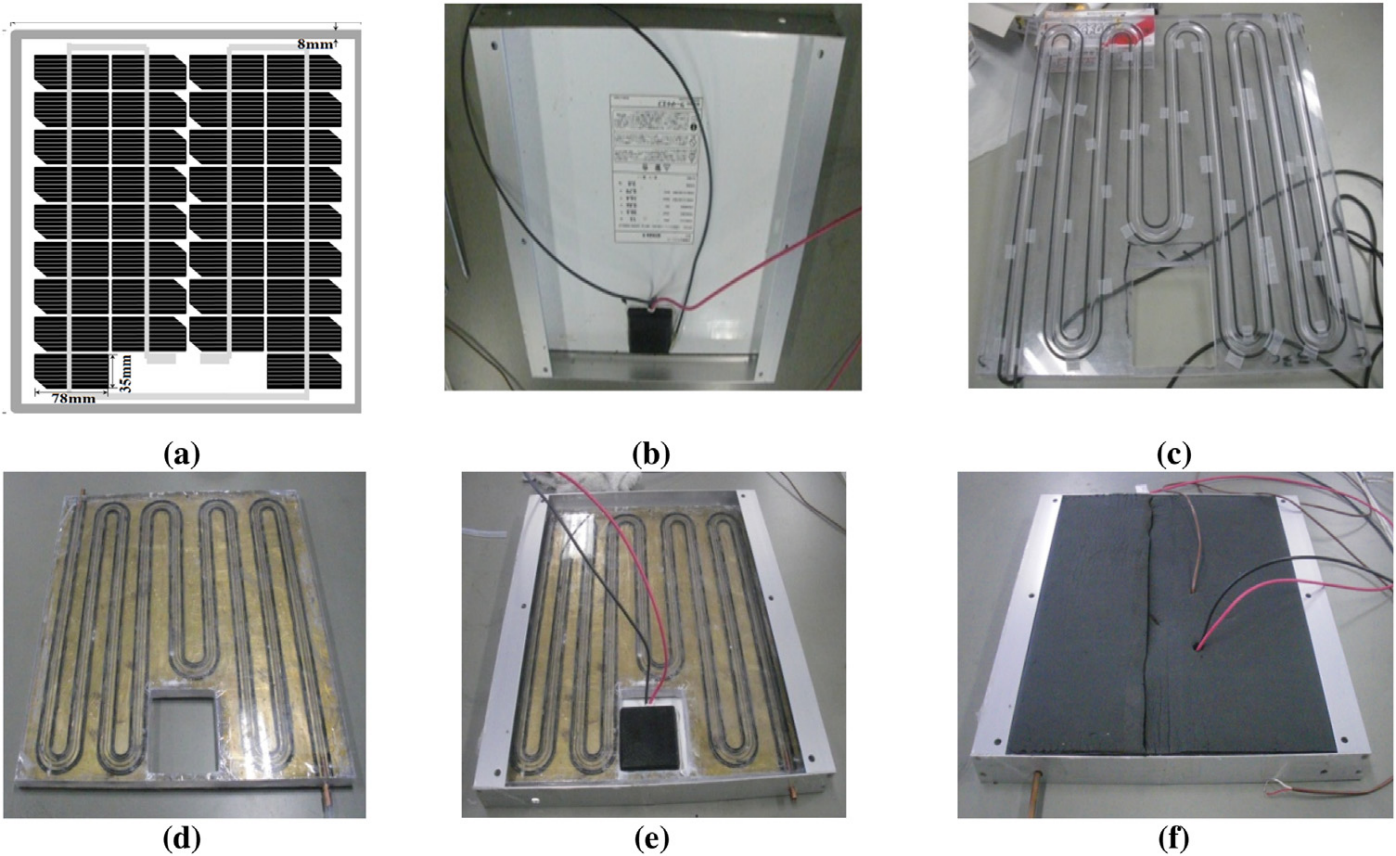
The assembly process of a polymeric PVT collector; a). The front side of PV-part, b). The rear side of PV-part, c). The sealed polymeric T-part, d). Assembly polymeric part with copper sheet absorber, e). T absorber inserted into PV-part before insulated, f). Rear side insulation of the polymeric PVT collector with thick rubber materials.

The purpose of this process is to combine PV-parts with T-parts to become PVT-collector. Fig. 3 shows the assembly process of a polymeric hybrid PVT collector. As shown in Fig. 3(a) is the front side. There is no modification for the front-side of the PV-part. Fig. 3(b) is the rear side of the PV-part. In preparation for the T-part to be easily inserted into the PV-part, the rear frame facing the wiring box must be removed and reinstalled. The adhesive rubber frames are peeled off little by little until they run out. Finally, the locking bolt is opened. The sealing rubber is installed to the polymeric T-part, as shown in Fig. 3(c). Fig. 3(d) shows the assembly process of the polymeric part with copper sheet absorber. The installation of bolts from the copper sheet to the polymeric absorber plate must be gradual and balanced, to reduce the risk of leakage. To keep the surface of the sealing rubber flat, the installation is done without pulling tightly. The T-part inserted into PV-part without giving silicon adhesive, before being insulated in Fig. 3(e) and rear side insulation with the thick rubber materials as shown in Fig. 3(f). Furthermore, the PVT collector was installed and tested with a solar simulator [44].

**Fig. 4:**
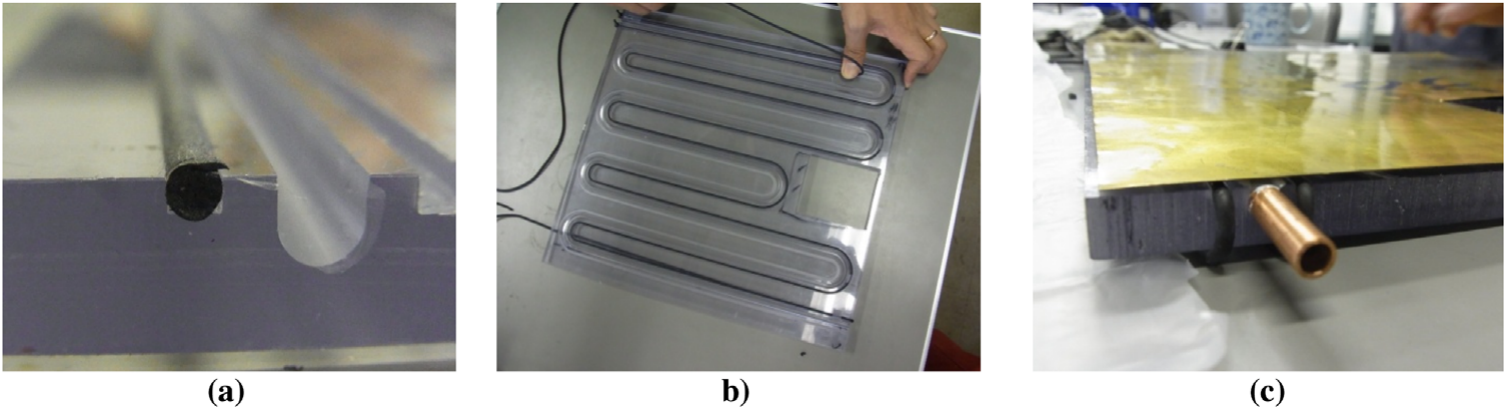
Sealing and channeling, a). Sealing rubber position, b). Installing sealing rubber, c). Exit pipe connection.

Fig. 4 shows a more detailed of the sealing and channeling of the T-part. In Fig. 4(a), it is clear that the difference between the rubber sealing channel and the water flow channel in terms of dimensions and position. As shown in the figure, the position of both channels are made closer to optimize the function of the channels in order to minimize the thermal energy losses. Fig. 4(b) shows the proper position of sealing rubber for the leak resistance. The process of mounting the rubber sealing into the sealing channel was done without pulling and without using glue. To hold the sealing rubber position during the installation process, a thin clear tape can be used. It is expected that the rubber packing will function optimally against leakage during thermal expansion. Fig. 4(c) shows the mounting position of the external copper pipe to be connected to the water hose (inlet and outlet). To anticipate the possibility of leakage, especially in the important part connection between the external copper pipe and the inlet and outlet of the PVT collector, the silicon glue was applied. Then, because these pipes are prone to be broken and loose, their position needs to be secured to avoid water leakage. As soon as the silicon glue dries, the hybrid polymeric PVT collector is ready to be installed into the solar simulator. The experiment can be started.

### Testing method

#### Set up of experiment

The purpose of this process is to prepare an experiment for PVT collector testing. For the experiment to run smoothly, some things need to be considered. All matters relating to the experiment must be prepared before the experiment is carried out, including calibration, reading of equipment, water conditions, and so on. If needed, we have to make a special checklist before the experiment, so that everything must be confirmed before experimenting.

**Fig. 5:**
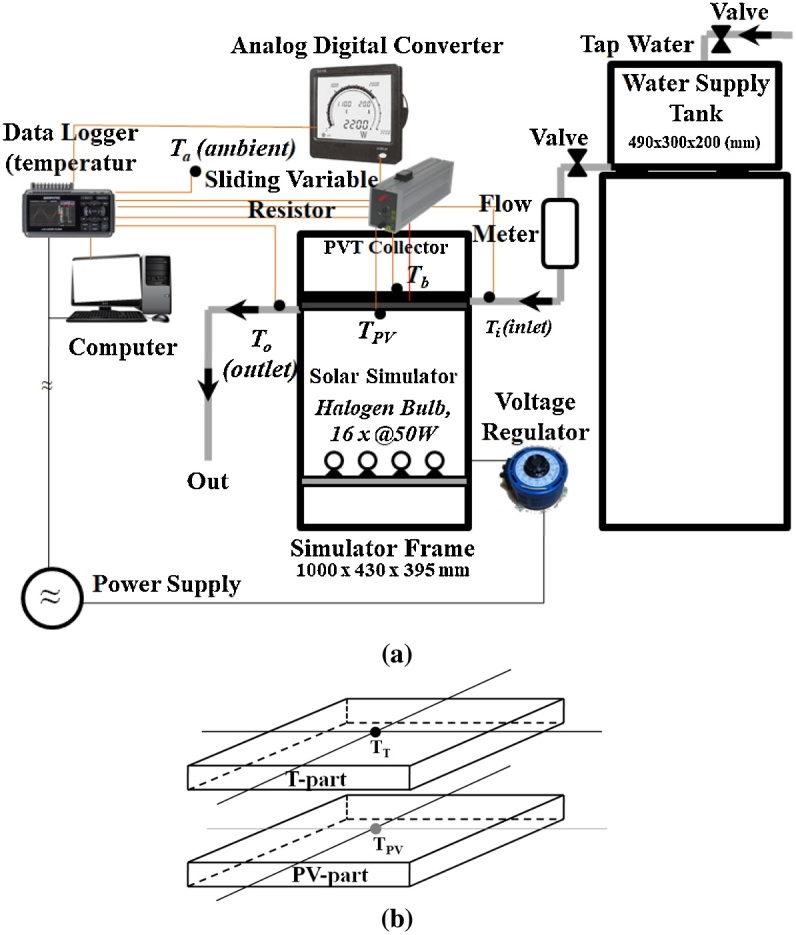
Experimental setup, a). Complete setup, b). Measurement position for $T_{pv}$ and.

Fig. 5 shows the complete setup and measurement of the experiments. As shown in Fig. 5(a), the irradiance could be changed by adjusting the voltage input $V_{i}$ to the halogen simulator using a sliding manual voltage regulator (Matsunaga Mfg, Co. Ltd, Type SD-1310) [44]. Here, we used tap water as a working fluid to absorb the heat from the PVT system that was stored in the water supply tank (WST). The water was not circulated in the simulator (open system). The water level in the WST has been controlled to maintain the stability of the water flowing into the PVT collector. The flow rate was measured by a floating ball type flow meter (111122-313, Kofloc) before and after the experiment and was also confirmed manually using a glass volume and stopwatch. The water temperature in the tank adjusts the tap water and room temperature. The temperature of inlet water $T_{i}$, outlet water $T_{o}$ and ambient air $T_{a}$ were measured by T-type thermocouples. The ambient air temperature $T_{a}$ was taken in the middle of the room. The PV module operating temperature here indicated by $T_{pv}$, plays a central role in the photovoltaic conversion process, where the power output of a PV module depends linearly on the operating temperature $T_{pv}$ [1]. In this case, considering the proper position and installation of the thermocouple is very important. This has a direct effect on the accuracy of the data to be collected. Before experimenting, all preparations had to be carefully examined and confirmed by the previous experiments. Then, the water hose must be protected so that it is not exposed directly to the light source from the simulator. The electric data of the PV, such as voltage (V), ampere (A), and power (W), were collected by an analogue to digital converter (WE1C, Fuji electric), sent to the data-logger (GL220, Graphtec), and then connected to a personal computer. The sampling time was set to 30 s.

As shown in Fig. 5(b), the surface temperature of PV, $T_{pv}$ was measured at the middle point on the rear side of the PV-part and the PVT back temperature, $T_{b}$, was measured at the middle point on the rear side of the T-part. The reason is to avoid the cable obstructing the light beam from the solar simulator. Before starting the experiment, the irradiance was measured using a pyranometer (MS-42, Eko). The distance between the PV surface and the pyranometer to the surface of light sources was set to 32 cm [44]. The PV output has been set at the maximum power point (MPP) by setting the sliding variable resistor. The input voltage of the voltage regulator is fixed. For the PV-part not to be exposed to halogen rays for too long, the experiment was carried out for 60 min and then the light source was turned off for cooling. After completely cooling$\left(T_{pv}\approx T_{a}\right)$, we started another experiment. It is important to ensure that the installation of $T_{pv}$ and $T_{b}$ thermocouple is correct, especially for $T_{pv}$. For both the PV-part and T-part surfaces to touch evenly, the $T_{pv}$ installation needs to be a little tricky. The trick, at the end of the $T_{pv}$ is made a little hollow and the cable is pulled up straight and continues to penetrate through the T-part. The position of $T_{b}$ is not far from the direction of the outgoing cable of $T_{pv}$. More details about the PV surface temperature distribution with electricity generation (PV-On) and without electricity generation (PV-Off), have been explained in [51].

**Fig. 6:**
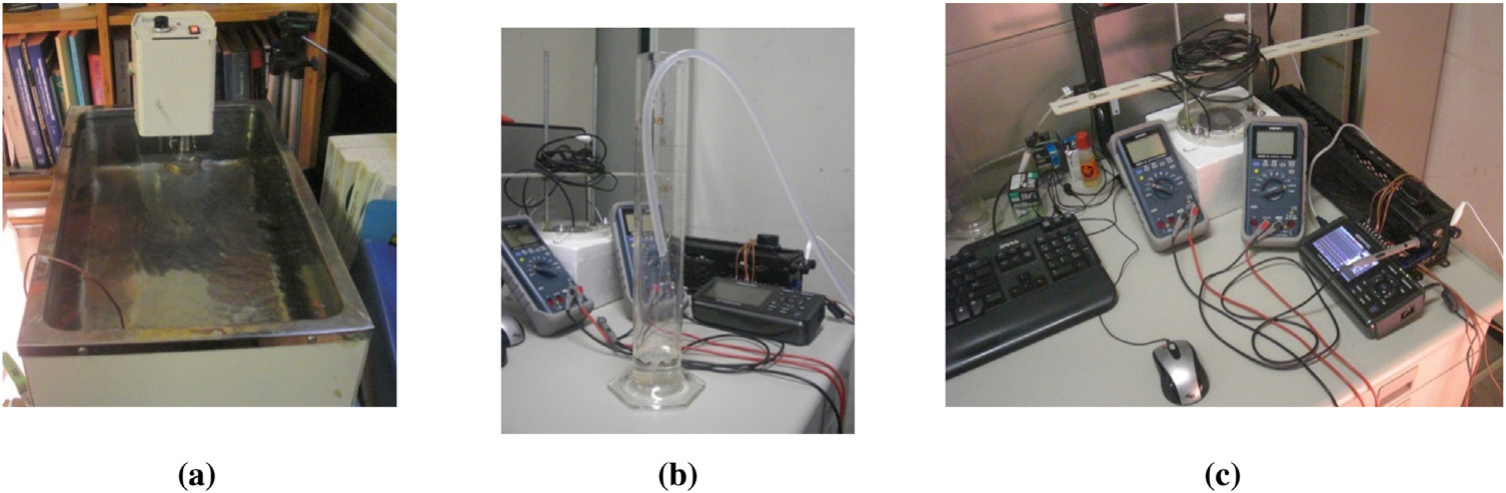
Experimental support, a). Water supply tank (WST), b). Manual glass volume, c). Data acquisition.

Fig. 6 shows the experimental support equipment used. Fig. 6(a) shows a water supply tank (WST), made of steel and has a heater and mixer. To keep the water condition normal without turbulence, the heater and mixer are not used. Fig. 6(b) shows a manual glass to measure the water volume used before experimenting, to ensure the flow rate is as expected. The method is simple, by pouring the water hose from the WST into the manual glass volume for a certain amount of time, assisted by a stopwatch. The mass flow rate is regulated using a water tap at WST. Fig. 6(c) shows the data acquisition and supporting equipment, both automatically and manually, such as data logger, digital multi-meters, pyranometer, sliding variable resistors, etc. The pyranometer is used before and after conducting experiments to ensure the expected irradiation stability of the solar simulator.

#### Theoretical thermal and electrical efficiency - calculation method

For more detail the thermal and electrical performance, we have conducted a series of experiments. The purpose of these experiments was to discover the effect of cooling on PV surface temperature $T_{pv}$ and also to electrical efficiency$\eta_{e}$, with fixed irradiance (I = 1000 W/m^2^), and variation of mass flow rate (${\dot{m}}_{w}$ = 200, 250, 300 g/min). In practical terms, the instantaneous thermal efficiency $\eta_{t}$ is defined as;

(3) $$\eta_{t}=\frac{Q_{u}}{Q_{s}}$$

where, the useful energy output, $Q_{u}$ [W], is given as ${\dot{m}}_{w}c_{p,w}\Delta T_{w}$ and the solar energy input, $Q_{s}$ [W] is given as $A_{pvt}I_{}$. Here, ${\dot{m}}_{w}$[g/min], $c_{p,w}$ [J/g.C], and $\Delta T_{w}$ [^o^C], are the mass flow rate, the specific heat of water, the temperature difference of water flowing in and out PVT collector, respectively. Then, Eq. (3) is rewritten as;

(4) $$\eta_{t}=\frac{{\dot{m}}_{w}c_{p,w}\Delta T_{w}}{A_{pvt}I}$$

Theoretically, the thermal efficiency of the solar collector, $\eta_{t}$, during steady-state can be expressed as [52]:

(5) $$\eta_{t}=F_{R}(\tau \alpha )-F_{R}U_{L}\frac{\left(T_{i}-T_{a}\right)}{I}$$

where, $F_{R}$ is the heat removal factor,τ is the transmittance,α is the absorptance, the product of τα is the optical efficiency $\eta_{o}$ $U_{L}$ is the collector overall loss coefficient [W/m^2^.°K], $T_{i}$ is water inlet temperature [°C], $T_{a}$ is the ambient air temperature [°C], and I represents the irradiation [W/m^2^]. From Eq. (1), high τα and low $U_{L}$ indicates that the collector system has high performance. To plot the graph in Eq.(5), we varied the irradiance $I_{}$ = 1000, 700, 400 W/m^2^. The heat removal factor$F_{R}$ is depend on the 3 dimensionless parameters, such as number of heat transfer unit N, collector efficiency factor F', and collector conduction factor K' [53]. Here, collector efficiency factor F' is strongly influenced by the distance between the tube or water channel and the thickness of absorber plate [54]. The collector overall loss coefficient $U_{L}$ is more influenced by the operational parameters, such as water mass flow rate ${\dot{m}}_{w}$ and inlet water temperature$T_{i}$ [55]. In practical terms, the electrical efficiency $\eta_{e}$ is also given by [31,56];

(6) $$\eta_{e}=\frac{V_{pv}I_{pv}}{A_{p}vI}$$

where, $V_{pv}$$I_{pv}$ and $A_{pv}$ represent as the voltage [V], the current [A] of PV with a particular load applied and PV module area [m^2^], respectively. Theoretically, $\eta_{e}$ can be expressed [1];

(7) $$\eta_{e}=\eta_{r}(1-\beta (T_{S}-T_{r}))$$

where β is the temperature coefficient of the efficiency of a crystalline PV module and is equal to 0.0045, $\eta_{r}$ is the reference efficiency of the PV cell at the reference temperature, $T_{r}$ (usually 25 °C) [57]. The overall efficiency of the PVT collector $\eta_{pvt}$ can be expressed as.

(8) $$\eta_{pvt}=\eta_{t}+\eta_{e}$$

Then, the PVT collector efficiency $\eta_{pvt}$ becomes optimal if T-part provides maximum thermal efficiency$\eta_{t}$, which from the cooling effect increases the electrical efficiency $\eta_{e}$ of PV-part.

#### Statistical calculation method

The standard deviation SD for the number of population samples n during the steady-state can be expressed [58];

(9) $$SD=\sqrt{\frac{\sum (\eta_{t,i}-{\bar{\eta }}_{t})}{n}}$$

To find out the accuracy of thermal efficiency data, the Standard Error SE can be performed using the following formula:

(10) $$SE=\frac{SD}{\sqrt{n}}$$

where; $\eta_{t,i}$ is the actual thermal efficiency value at a certain point during the steady-state and ${\bar{\eta }}_{t}$ is the average value of the actual thermal efficiencies $\eta_{t,i}$ during the steady-state.

### Result and discussion

**Fig. 7:**
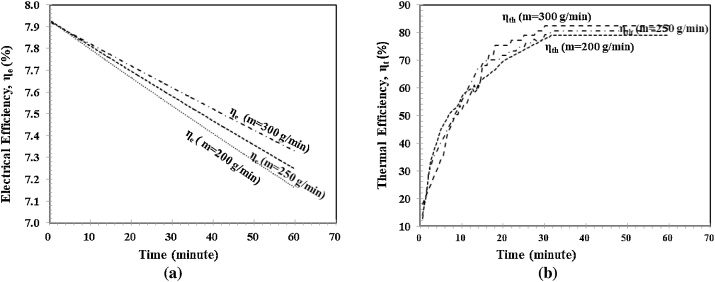
Electrical and thermal performance, a) Electrical efficiency $\eta_{e}$ vs Time, 2). Thermal efficiency $\eta_{t}$vs Time.

Fig. 7 shows the cooling effect of different mass flow rates ${\dot{m}}_{w}$ to the electrical and thermal performance of the hybrid polymeric PVT collector against the time. As shown in Fig. 7(a), the cooling effects of the different mass flow rates ${\dot{m}}_{w}$ provide the different electrical efficiency $\eta_{e}$. With the larger mass flow rates ${\dot{m}}_{w}$, the cooling effect is also larger. As a result, the decrease in electrical efficiency $\eta_{e}$ becomes smaller. At the beginning of the experiment, the electrical efficiencies $\eta_{e}$ were started at the same point; 7.92%. After 60 min, they were at the different points, namely; 7.4%, 7.3%, 7.2% for mass flow rates ${\dot{m}}_{w}$ of 300, 250, 200 g/min, respectively. For Fig. 7(b), the cooling effect by a different mass flow rate ${\dot{m}}_{w}$ gives a different effect to the thermal efficiency $\eta_{t}$. Larger mass flow rates ${\dot{m}}_{w}$ provide a higher thermal efficiency $\eta_{t}$ compared to the smaller mass flow rates ${\dot{m}}_{w}$. Besides, the graph also shows that the steady-state is reached in about 30 min for all three mass flow rates ${\dot{m}}_{w}$. At the beginning of the experiment, the thermal efficiencies $\eta_{t}$ were at the same point; 0%. After 60 min, they were at different points, around 80%, the higher mass flow rate provide a little higher thermal efficiency.

**Fig. 8:**
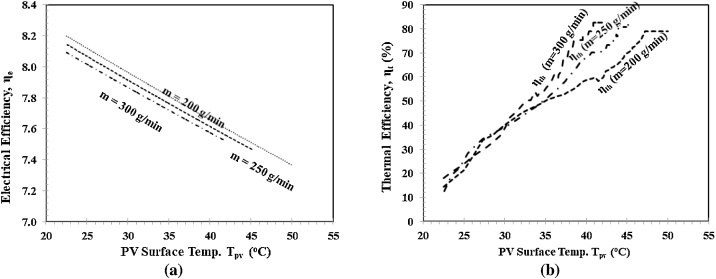
Electrical and thermal performance, a) Electrical efficiency $\eta_{e}$ vs $T_{pv}$, 2). Thermal efficiency $\eta_{t}$vs.

Fig. 8 shows the cooling effect of different mass flow rates ${\dot{m}}_{w}$ to the electrical and thermal performance of the hybrid polymeric PVT collector against the PV surface temperature $T_{pv}$. Fig. 8(a) shows the cooling effects of different mass flow rates ${\dot{m}}_{w}$ to the electrical efficiency $\eta_{e}$ against the PV surface temperature $T_{pv}$. As shown, with larger mass flow rates ${\dot{m}}_{w}$, the cooling effect is also larger. As a result, with the same PV surface temperature $T_{pv}$, the decrease in electrical efficiency $\eta_{e}$ becomes smaller. At the beginning of the experiment, electrical efficiency $\eta_{e}$ for the three mass flow rates ${\dot{m}}_{w}$ were started at the same point; 7.92%. After 60 min, they were at different points of PV surface temperatures $T_{pv}$, namely; 50, 45, 40 ^0^C for mass flow rates ${\dot{m}}_{w}$ of 200, 250, 300 g/min, respectively. Fig. 8(b) shows the cooling effects of different mass flow rates ${\dot{m}}_{w}$ to the thermal efficiency $\eta_{t}$ against the PV surface temperature $T_{pv}$. As shown, larger mass flow rates ${\dot{m}}_{w}$ provide a higher thermal efficiency $\eta_{t}$ compared to the smaller mass flow rates ${\dot{m}}_{w}$. Besides, the graph also shows that thermal efficiency $\eta_{t}$ is still the same for all mass flow rates ${\dot{m}}_{w}$ before the steady-state around 30 min. After that, a larger mass flow rate ${\dot{m}}_{w}$ also shows larger thermal efficiency $\eta_{t}$. At the beginning of the experiment, the thermal efficiency $\eta_{t}$ for the three mass flow rates ${\dot{m}}_{w}$ was at the same point; 0%. After 60 min, they were at different points, around 80%, with the different PV surface temperature; 50, 45, 40 ^0^C for mass flow rates ${\dot{m}}_{w}$ of 200, 250, 300 g/min, respectively.

**Fig. 9:**
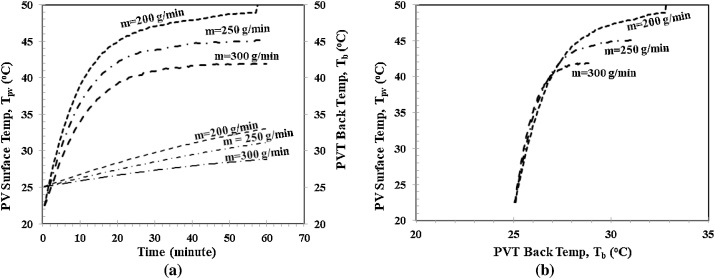
PV surface and PVT back temperature, a) $T_{pv}$and $T_{b}$vs Time, b). $T_{pv}$vs.

Fig. 9 shows the cooling effect of different mass flow rates ${\dot{m}}_{w}$ to the PV surface temperature $T_{pv}$ and the PVT back temperature$T_{b}$. Fig. 9(a) shows the cooling effects of different mass flow rates to the PV surface temperature $T_{pv}$ and the PVT back temperature $T_{b}$ against time. As shown, with larger mass flow rates ${\dot{m}}_{w}$, the cooling effect is also larger. As a result, with the same period, the PV surface temperature $T_{pv}$ and PVT back temperature $T_{b}$ become smaller. After steady-state, both $T_{pv}$ and $T_{b}$ become flattered for a larger mass flow rate. Fig. 9(b) shows the cooling effect of different mass flow rate ${\dot{m}}_{w}$ in the correlation between the PV surface temperature $T_{pv}$ and the PVT back temperature $T_{b}$. As shown, larger mass flow rates ${\dot{m}}_{w}$ provide a higher cooling effect thermal efficiency $\eta_{t}$ with lower $T_{pv}$ and $T_{b}$.

**Fig. 10:**
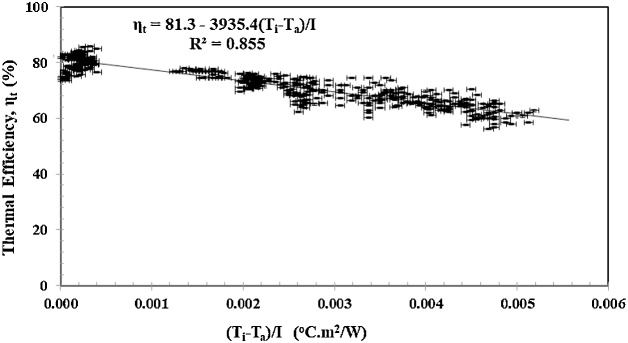
Thermal efficiency$\eta_{t}$ vs Reduced temperature.

Fig. 10 illustrates the thermal efficiency $\eta_{t}$ against the reduced temperature $(T_{i}-T_{a})/I$, based on experiments with variations in mass flow rates $\left(\dot{m}=300,\text{ 250, 200 g/m}\right)$and irradiances $\left(I=1000,\text{ 700, 400 W/}{\text{m}}^{2}\right)$. Referring to Eq. (5), Fig. 5 shows that the $F_{R}(\tau \alpha )$ is 0.813, while the $F_{R}U_{L}$ is 39.35. Assuming τα = 0.9 [59], then $F_{R}$ and $U_{L}$ can be roughly estimated to be 0.9 and 43.7 W/m^2^.K, respectively. At the zero reduced temperature $(T_{i}-T_{a})/I=0$, or when the inlet and outlet temperatures are the same, $T_{i}=T_{a}$, the thermal efficiency $\eta_{t}$ is the same as $F_{R}\tau \alpha$, or 0.813. The standard deviation values SD based on Eq. (9) and the standard error SE based on Eq. (10) are 6.8% and 0.30, respectively. Both SD and SE values are quite large due to the sharp gradient of the thermal efficiency $\left(F_{R}U_{L}=39.35\right)$. For this reason, we replaced the average value of actual thermal efficiency with the regression value of thermal efficiency. As a result, SD and SE become 0.30 and 0.11. Fig. 10 also illustrates that the thermal efficiency will be higher if the inlet water temperature approaches the ambient air temperature, or getting closer to the zero reduced temperature $(T_{i}-T_{a})/I\approx 0$.

Based on what we have explained about the method development and testing of the polymeric PVT collector, at least there are two interesting things to discuss.

*First,* regarding the development method of the polymeric PVT collector. The polymeric PVT collector can be made easily, using simple tools with just average skills. However, some things need to be considered. In the initial stages of design, things like functions, applications, costs, complexity, etc. of the collector PVT must be clear. Following its function for indoor experiments with low cost, we made it manually, without welding and pressing process, between PV-part and T-part can be separated to experiment with/without cooling, etc.

*Second,* regarding the testing method of the polymeric PVT collector. The testing process is very important to understand and implement carefully so that the PVT collector functions properly with optimal performance. For this reason, the important parameters such as the mass flow rate and inlet water temperature must be anticipated from the beginning, as expressed theoretically in [52] (Eq.6.17.5). In theoretical terms, the electrical efficiency of the PV module, which is dependent on the PV temperature $T_{pv}$, can be expressed in [60,61]. The failures to anticipate those parameters will cause ineffective cooling effects given by the working fluids, as can be seen from the increase in PV surface temperature that is too large or too small.

*Third,* regarding the overall performance of the polymeric PVT collector. With the thermal efficiency reaching more than 81%, which has a positive impact on electrical efficiency through its cooling effect, the hybrid polymeric PVT collector is quite promising. Then, referring to the high heat removal factor $F_{R}$ based on the estimated values for optical efficiency, it can be assumed that the current polymeric hybrid PVT collector is quite appropriate for its geometrical design.

The practical contribution of this method is how a hybrid PVT collector can be made easily and simply for an indoor scale, even for a household scale. Besides, this method can be applied not only to water as working fluid, but also to air as a working fluid for hybrid polymeric PVT collector, as long as the size of the collector is still possible to be made manually with those simple tools. For larger collectors, it can still be made modularity connected in series to be a larger one. Then, each module must be tested for performance with the same standard and certain quality tolerances. For the future direction, it is necessary to consider how to reduce the thermal resistance from PV-part to T-part, for example; to eliminate the copper plate by connecting the PV-part directly to the T-part, reducing the mass of the T-part with a thinner one, etc.

## Conclusion

We have explained the method development and testing of the polymeric PVT collector from the design, fabrication/integration to the performance testing. The main results are summarized as follows:

• The polymeric hybrid PVT collector can be made with simple methods, tools, and materials for the indoor experiment purposes.
• PMMA as a polymer material can be used as a thermal absorber or T-part for a hybrid PVT collector with a machining process without any welding process.
• The performance of the polymeric hybrid PVT collector can provide optimal cooling effects to PV surface temperature, thus providing optimum electrical and thermal efficiency.
• With a high value of heat removal factor $F_{R}$, indicating that the geometrical design of this hybrid polymeric PVT collector is quite appropriate.
• This manual method can still be applied to make polymeric hybrid PVT collectors with water as the working fluid but also for air, even for the larger ones with modular systems.
• For the future direction, it is necessary to consider how to reduce thermal resistance from PV-part to T-part, by connecting PV-part directly to the T-part, reducing the mass of the T-part with a thinner one, etc.

## Declaration of Competing Interest

The author declares that there is no conflict of interest regarding the publication of this article.
